# A scuticociliate causes mass mortality of *Diadema antillarum* in the Caribbean Sea

**DOI:** 10.1126/sciadv.adg3200

**Published:** 2023-04-19

**Authors:** Ian Hewson, Isabella T. Ritchie, James S. Evans, Ashley Altera, Donald Behringer, Erin Bowman, Marilyn Brandt, Kayla A. Budd, Ruleo A. Camacho, Tomas O. Cornwell, Peter D. Countway, Aldo Croquer, Gabriel A. Delgado, Christopher DeRito, Elizabeth Duermit-Moreau, Ruth Francis-Floyd, Samuel Gittens, Leslie Henderson, Alwin Hylkema, Christina A. Kellogg, Yasunari Kiryu, Kimani A. Kitson-Walters, Patricia Kramer, Judith C. Lang, Harilaos Lessios, Lauren Liddy, David Marancik, Stephen Nimrod, Joshua T. Patterson, Marit Pistor, Isabel C. Romero, Rita Sellares-Blasco, Moriah L. B. Sevier, William C. Sharp, Matthew Souza, Andreina Valdez-Trinidad, Marijn van der Laan, Brayan Vilanova-Cuevas, Maria Villalpando, Sarah D. Von Hoene, Matthew Warham, Tom Wijers, Stacey M. Williams, Thierry M. Work, Roy P. Yanong, Someira Zambrano, Alizee Zimmermann, Mya Breitbart

**Affiliations:** ^1^ Department of Microbiology, Cornell University, Ithaca, NY, USA.; ^2^ College of Marine Science, University of South Florida, St. Petersburg, FL, USA.; ^3^ U.S. Geological Survey, St. Petersburg Coastal and Marine Science Center, St. Petersburg, FL, USA.; ^4^ School of Forest, Fisheries, and Geomatics Sciences, Institute of Food and Agricultural Sciences, University of Florida, Gainesville, FL, USA.; ^5^ Emerging Pathogens Institute, University of Florida, Gainesville, FL, USA.; ^6^ Department of Planning and Natural Resources, Virgin Islands Government, Christiansted, VI, USA.; ^7^ National Coral Reef Management Fellowship, Nova Southeastern University, Fort Lauderdale, FL, USA.; ^8^ Center for Marine and Environmental Studies, University of the Virgin Islands, Saint Thomas, VI, USA.; ^9^ Antigua and Barbuda National Parks Authority, Nelson’s Dockyard, Antigua and Barbuda.; ^10^ St Eustatius National Parks Foundation, Oranjestad, Caribbean, Netherlands.; ^11^ Bigelow Laboratory for Ocean Sciences, East Boothbay, ME, USA.; ^12^ Central Caribbean Program, The Nature Conservancy, Santo Domingo, Dominican Republic.; ^13^ Florida Fish and Wildlife Conservation Commission, Fish and Wildlife Research Institute, Marathon, FL, USA.; ^14^ College of Veterinary Medicine, University of Florida, Gainesville, FL, USA.; ^15^ National Oceanic and Atmospheric Administration Office for Coastal Management, Silver Spring, MD, USA.; ^16^ Van Hall Larenstein University of Applied Sciences, Leeuwarden, Netherlands.; ^17^ Marine Animal Ecology Group, Wageningen University, Wageningen, Netherlands.; ^18^ Florida Fish and Wildlife Conservation Commission, Fish and Wildlife Research Institute, St. Petersburg, FL, USA.; ^19^ Caribbean Netherlands Science Institute, St. Eustatius, Caribbean, Netherlands.; ^20^ NIOZ Royal Netherlands Institute for Sea Research, Oranjestad, Caribbean, Netherlands.; ^21^ Ocean Research and Education Foundation, Atlantic and Gulf Rapid Reef Assessment, Big Pine Key, FL, USA.; ^22^ Smithsonian Tropical Research Institute, Panama City, Republic of Panama.; ^23^ Montclair State University, Montclair, NJ, USA.; ^24^ School of Veterinary Sciences, St. George’s University, St. George’s, Grenada.; ^25^ Department of Biology, Ecology and Conservation, St. George’s University, St. George’s, Grenada.; ^26^ Fundación Dominicana de Estudios Marinos, Bayahibe, Dominican Republic.; ^27^ Saba Conservation Foundation, Saba, Caribbean, Netherlands.; ^28^ Institute for Socio-Ecological Research, Lajas, Puerto Rico.; ^29^ U.S. Geological Survey, National Wildlife Health Center, Honolulu Field Station, Honolulu, HI, USA.; ^30^ Red Arrecifal Dominicana, Santo Domingo, Dominican Republic.; ^31^ Turks and Caicos Reef Fund, Providenciales, Turks and Caicos Islands.

## Abstract

Echinoderm mass mortality events shape marine ecosystems by altering the dynamics among major benthic groups. The sea urchin *Diadema antillarum*, virtually extirpated in the Caribbean in the early 1980s by an unknown cause, recently experienced another mass mortality beginning in January 2022. We investigated the cause of this mass mortality event through combined molecular biological and veterinary pathologic approaches comparing grossly normal and abnormal animals collected from 23 sites, representing locations that were either affected or unaffected at the time of sampling. Here, we report that a scuticociliate most similar to *Philaster apodigitiformis* was consistently associated with abnormal urchins at affected sites but was absent from unaffected sites. Experimentally challenging naïve urchins with a *Philaster* culture isolated from an abnormal, field-collected specimen resulted in gross signs consistent with those of the mortality event. The same ciliate was recovered from treated specimens postmortem, thus fulfilling Koch’s postulates for this microorganism. We term this condition *D. antillarum* scuticociliatosis.

## Teaser

A scuticociliate (*Philaster *sp.) causes mass mortality of the long-spined sea urchin *Diadema antillarum*.

## INTRODUCTION

Mass mortality events have wide-reaching effects on marine ecosystem function and food web structure (*1*). The long-spined sea urchin, *Diadema antillarum*, experienced mass mortality in the Caribbean from 1983 to 1984, leading to declines of ~98% compared to premortality population densities (*2*–*5*). Ecologically functional *D. antillarum* population recovery was prolonged and estimated at ~12% of pre-event densities 30 years later (*5*). The loss of this herbivore contributed to a change in the competitive relationships between stony corals and benthic algae. In conjunction with other stressors, this mass mortality led to the rapid degradation of many coral reefs across the region (*6*, *7*). The cause of the 1980s *D. antillarum* mass mortality was never determined, and no specimens of affected urchins from that time exist in museum collections or other sample repositories. However, the spread of the mass mortality in the 1980s was consistent with the hypothesis that the causative agent was dispersed by water currents over long distances (*3*). Molecular biological approaches for the study of potential pathogens were not available at the time. Some urchin and other echinoderm mass mortality events have been ascribed to potential pathogens, but they have poorly understood etiologies that center on environmental stress, microbial dysbiosis, or some combination of these (*8*–*11*). Koch’s postulates (*12*) have rarely been fulfilled for the few microorganisms proposed as associates of disease in echinoderms or any other marine invertebrate (*13*, *14*).

**Fig. 1.**
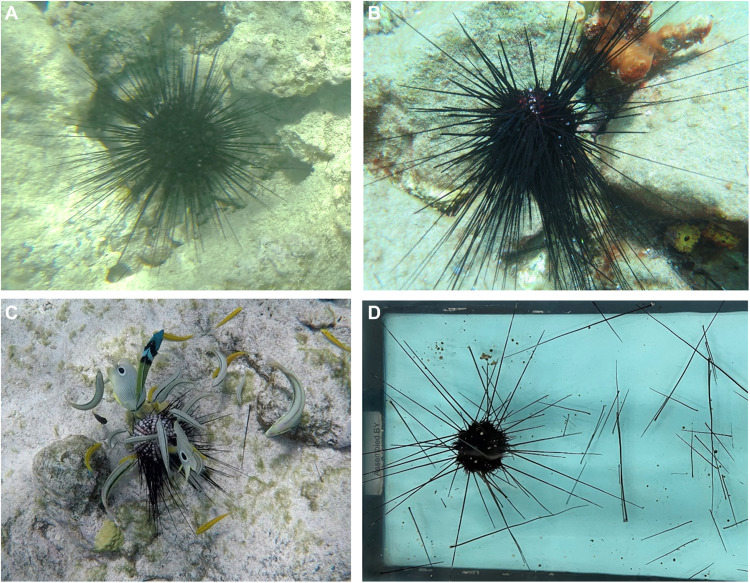
Progression of abnormal condition affecting *D. antillarum*. (**A** to **D**) Grossly normal specimens (A) exhibit upright posture and typically shelter beneath rock/coral (photo taken in Pope Point, St. John, April 2022). At condition onset, specimens fall over or stand on spines and exhibit drooping spines (B) (photo taken in Saba, Caribbean Netherlands, April 2022). Advanced stages present as spine loss with elevated predation by fish (C) (photo taken in Aruba, August 2022) and, eventually, death. These signs were also observed in specimens that were experimentally challenged with the *Philaster* culture FWC2 in aquaria (D). Photo credits: (A) and (D) I. Hewson, (B) A. Hylkema, and (C) D. Behringer.

In late January 2022, *D. antillarum* began experiencing another mass mortality event. The condition was first observed in St. Thomas [U.S. Virgin Islands (USVI)] and, by late March, had been found at nine more insular Caribbean jurisdictions across the Lesser Antilles, Jamaica, and the Mexican Caribbean (*15*). By June 2022, the condition had also been reported in most of the Greater Antilles, Florida, and Curaçao (*15*). Gross signs in affected urchins began with detachment from vertical surfaces, loss of spine movement/reaction to stimuli, loss of tube foot control, using spines for locomotion instead of tube feet, and forming a stellate spine arrangement (Fig. 1). Within days, urchins with these signs catastrophically lost spines leading to epidermal tissue loss and exposure of underlying test which progressed rapidly (~2 days) to death (*15*). The loss of spines, in particular, led to predation by nearby fishes (Fig. 1C) or invertebrates. At sites in the USVI, large masses of turbid water accompanied the onset of the condition, and in at least four other sites (northern Puerto Rico, Aruba, Saba, and St. Eustatius), heavy rainfall preceded mass mortality. Large rafts of shoaling *Sargassum* spp. were observed at some affected sites. However, at other sites, no abnormal environmental conditions were noted (e.g., Jamaica and Dominican Republic). We investigated the potential drivers of this condition through complementary histopathological, cytological, and molecular biological approaches by comparing three types of *D. antillarum*: grossly normal and abnormal specimens collected from affected sites and grossly normal specimens from sites where the condition was not present (i.e., reference sites; see the Supplementary Materials).

## RESULTS

For the molecular biological approach, we investigated the phenomenon initially through holobiont (i.e., the urchin and its associated microorganisms) transcriptomes of the coelomic fluid and body wall. Early specimens for this approach were collected at Saba (Caribbean, Netherlands) and St. John (USVI). Transcriptomes comparing the three specimen types did not reveal any viruses, archaea, or bacteria uniquely associated with abnormal tissues (fig. S1). A total of 12 differentially expressed genes (DEGs) were ribosomal RNAs (rRNAs) of eukaryotes and bacteria (fig. S1). Of the remaining 59 DEGs, most could be characterized as retrotransposons or endogenized retroviruses (*n* = 19), genes involved in replication and repair (*n* = 10), and genes involved in signal transduction and repair (*n* = 8) functions. Comparison of expression values among functional pathways indicated the enrichment of most DEG pathways in abnormal urchins compared to grossly normal urchins from affected and reference sites (fig. S2). However, DEGs involved in cytoskeleton structure were highly depressed in abnormal urchins relative to grossly normal urchins from affected and reference sites. Notably, genes involved in complementation (predominately macroglobulin) had the greatest enrichment in abnormal urchins.

**Fig. 2.**
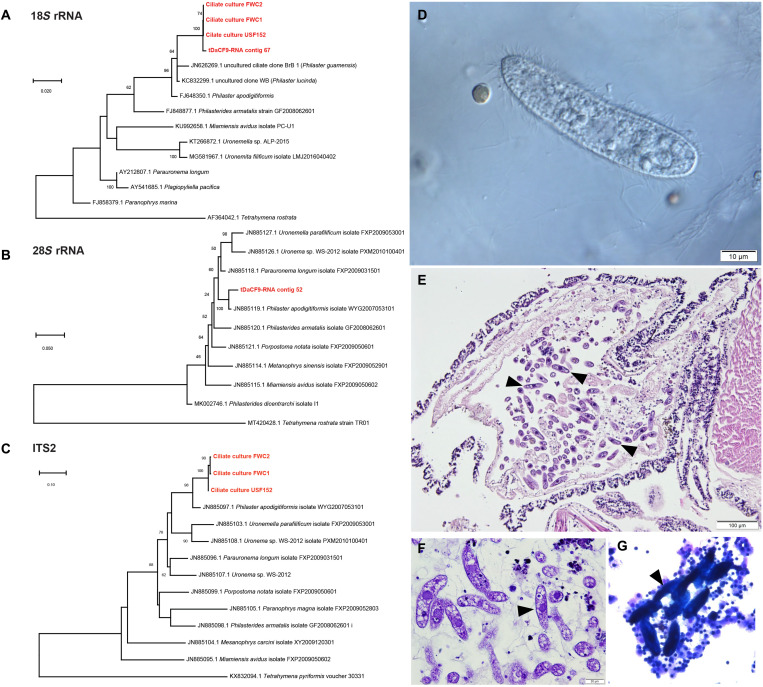
Phylogenetic and microscopic analyses of *Philaster *sp. in abnormal *D. antillarum*. (**A** to **C**) Phylogenetic representation of 18S rRNA and 28S rRNA Philaster-like sequences recovered from transcriptomes and 18S rRNA and ITS2 amplified from cultures FWC1 and FWC2 (cultured from abnormal urchins from an affected patch reef off Key Largo, Florida) and USF152 (cultured from an affected urchin during the experimental challenge study).These sequences are compared to homologous sequences of related species obtained from GenBank (accession numbers precede species names). Trees were constructed by maximum likelihood, the Jukes-Cantor substitution model, and with uniform rates of substitution. Bootstrap values of >50 (from 100 replicates) are indicated at nodes. Ciliates with morphologies similar to described *Philaster* sp. were observed by wet mount light microscopy (Nomarski prism) (**D**), in the base portion of the spine shaft by histopathology [indicated by arrowheads in (**E**) hematoxylin and eosin and (**F**) thionin], and in stained coelomic fluid cytology [indicated by arrowheads in (**G**); DiffQuick]. Photo credits: (D) to (F) Y. Kiryu and (G) T. Work.

Seventeen contigs of the complete contig spectrum (*n* = 51,691) matched bacterial 16*S* and 23*S* rRNA genes, of which 12 recruited >10 reads total. These represented Pseudomonadota (*n* = 5), Bacteroidota (*n* = 4), Fusobacteriota (*n* = 2), and Bacillota (*n* = 1). Apart from the Bacillota, which was distantly [91% across 327 nucleotides (nt)] related to a sequence recovered from lake water, the remaining sequences most closely matched 16*S* rRNAs recovered from animal tissues (*n* = 8), coral surfaces (*n* = 1), coral mucus (*n* = 1), and macroalgae (*n* = 1). Recruitment patterns revealed that all but 1 of these 12 contigs (a *Thiotrichales*-like bacterium) were less well-represented in abnormal urchin libraries than in grossly normal urchin libraries from affected or reference sites. After removing sequences matching urchin 18*S* and 28*S* rRNAs, the complete contig spectra also yielded 28 contigs that matched nonurchin eukaryotic rRNAs (a diatom, a dinoflagellate, a fungus, six ciliates, and seven apicomplexans). Of these, only two ciliate contigs had significantly (Mann-Whitney *U* test, *P* = 0.017 and *P* = 0.009, respectively) greater representation in abnormal urchins relative to either grossly normal urchins at the reference site or to both grossly normal urchins at the reference site and the grossly normal urchins at the affected site combined. Ciliates are well known to occur as symbionts in grossly normal urchins (*16*). However, sequences most similar (99% nucleotide ID against the 28*S* rRNA, 99% against the Internally Transcribed Spacer 2 (ITS2) region, and 92% against the 18*S* rRNA) to a pathogenic scuticociliate closely related to *Philaster apodigitiformis* (*17*) were observed (GenBank accession numbers OP896969 and OP962296). The *P. apodigitiformis* 18*S* rRNA sequences were most similar to uncultivated scuticociliate sequences [*Philaster guamense* and *Philaster lucinda* (*18*–*20*)] recovered from two corals affected by brown band and white band diseases and white syndrome (*21*, *22*). In addition, light microscopic examination of coelomic fluid of affected urchins revealed cells consistent in morphology with scuticociliate taxa (Fig. 2G) (*22*, *23*). 

**Fig. 3.**
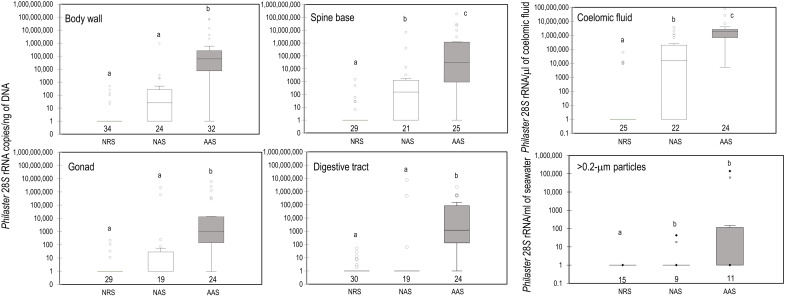
Box plots of *Philaster*-like 28*S* rRNA loads determined by qPCR for different sample types (body wall, spine base, coelomic fluid, gonad, digestive tract, and >0.2-μm particles collected immediately above urchins) acquired from different specimen types. The number of specimens surveyed is indicated below box plots. a, b, and c denote groups that are not statistically different (Kruskal-Wallis test with Dunn’s post hoc correction for multiple comparisons). The AAS box plots are shaded gray to highlight results from abnormal specimens. NRS, grossly normal at reference site; NAS, grossly normal at affected site; AAS, abnormal at affected site.

We designed and validated a quantitative polymerase chain reaction (qPCR) assay targeting the scuticociliate 28*S* rRNA gene and applied this assay to DNA extracts of spine base, coelomic fluid, aboral and dorsal body wall, digestive tract, and gonad tissues from all urchins surveyed from 23 sampling sites across the Caribbean (see the Supplementary Materials) (Fig. 3). The *Philaster*-like ciliate DNA was highly enriched in grossly abnormal specimens compared to grossly normal specimens from the same affected site and was absent from specimens at reference sites. The ciliate was observed in all grossly abnormal and some grossly normal specimens collected from Brewer’s Bay, St. Thomas, in February 2022, which was among the earliest observations of mass mortality during the current event (fig. S3 and table S2). For all abnormal specimens, the greatest ciliate abundance occurred within spine base tissues and body wall tissues, while the ciliate was rarely detected in the digestive tract or gonad samples. The rare detection in the digestive tract and gonad samples of abnormal urchins suggests that the ciliate is an external infection and not overgrowth due to ante mortem stress or postmortem spread. At St. John (USVI), we also detected the *Philaster*-like ciliate in particulate material (>0.2 μm) from seawater collected within 50 cm of abnormal specimens (5 of 11 samples tested) and sporadically in sediments collected nearby (two of nine samples tested). These data suggest a solid numerical association between *D. antillarum* mass mortality and the presence and load of the *Philaster*-like ciliate across the Caribbean between February and June 2022.

Next, we cultivated the *Philaster*-like ciliate from affected urchins. Coelomic fluid was taken from two grossly abnormal urchins that had been collected from an affected patch reef off Key Largo (Florida, USA, 24.9508°N, 80.4904°W) on 15 June 2022 (note that this was a reference site on 7 May 2022) and immediately amended to a ciliate cultivation medium (table S2). Observation of cultures 24 hours after collection revealed the presence of a ciliate with highly similar morphology to the ciliate cells observed in preserved coelomic fluid samples from abnormal urchins (cells that were absent in grossly normal specimens). Dilution-to-extinction subculture generated xenic culture with uniform morphology, termed FWC2. DNA extracts from the cultures generated qPCR amplicons, and direct amplification by PCR across two loci (ITS2 and 18*S *rRNA) revealed sequences that were near-identical (99% nucleotide identity across 945 nt) to transcriptome-assembled rRNAs (GenBank accession numbers OP896845 to OP896853; Fig. 2, A to C). Amendment of the subsequent subculture of this dilution with 0.2-μm filtered aboral body wall tissue homogenate (1 g of tissue in 10 ml of sterile seawater) resulted in considerable growth. Amendment of unfiltered body wall tissue homogenate likewise stimulated the growth of the ciliate above both unamended and 0.2-μm tissue homogenate amendments (fig. S4). These results show that the *Philaster*-like ciliate thrives on cellular material derived from *Diadema* tissues.

Histopathology and coelomic fluid cytology revealed the presence of ciliates with morphology consistent with Scuticociliata in field-collected specimens (tables S1 and S2). Coelomic fluid observations in 15 specimens (*n* = 4 grossly normal from reference sites, *n* = 5 grossly normal from affected sites, and *n* = 6 grossly abnormal from affected sites) revealed the presence of a scuticociliate-like protist in three smears from abnormal urchins (50%), compared to one each from grossly normal urchins at affected sites (20%) and grossly normal urchins at reference sites [25%; Fisher’s exact test, *P* = 0.65, not significant (n.s.)]. Cytology also revealed the presence of at least two additional ciliate morphotypes and three unidentified unicellular eukaryotes. These were found less frequently in grossly abnormal urchin specimens (33%) compared to grossly normal urchins from both affected and reference sites, although the differences were not statistically significant (collectively 55%; Fisher’s exact test, *P* = 0.88, n.s.). Filamentous and coccoid bacteria were also observed in two of six grossly abnormal urchins and in one of nine grossly normal urchins. Ciliates with morphology consistent with *Philaster* spp. were consistently observed in tube foot tissue (observed in wet mount exams; Fig. 2D) from field-collected abnormal urchins [14 of 18 (78%)], compared to grossly normal urchins from affected sites [1 of 11 (9%); Fisher’s exact test, *P* = 0.0015] (table S1). Notably, scuticociliates were not observed in tube foot tissue from any of the 24 specimens from reference sites (0%; Fisher’s exact test compared to abnormal urchins, *P* < 0.0001). However, there was no difference between the presence of scuticociliates observed in tube foot tissues in grossly normal urchins from affected sites and reference sites (Fisher’s exact test, *P* = 0.9429, n.s.). Scuticociliates were also observed across all histological thin sectioned tissues, especially in the spines, tube feet, ampullae, and other organs (Fig. 2, E and F) in 28 of 28 (100%) abnormal urchins, 6 of 20 (30%) grossly normal urchins from affected sites, and 1 of 30 (3%) grossly normal urchins from reference sites (Fisher’s exact test between all comparisons, *P* < 0.05). Overall, these data are consistent with molecular observations associating *D. antillarum* mass mortality with the presence of scuticociliates in tissues and coelomic fluids. The presence of scuticociliates in grossly normal specimens from affected sites may reflect preclinical infection (i.e., before animals become grossly abnormal) or resistant host genotypes.

**Fig. 4.**
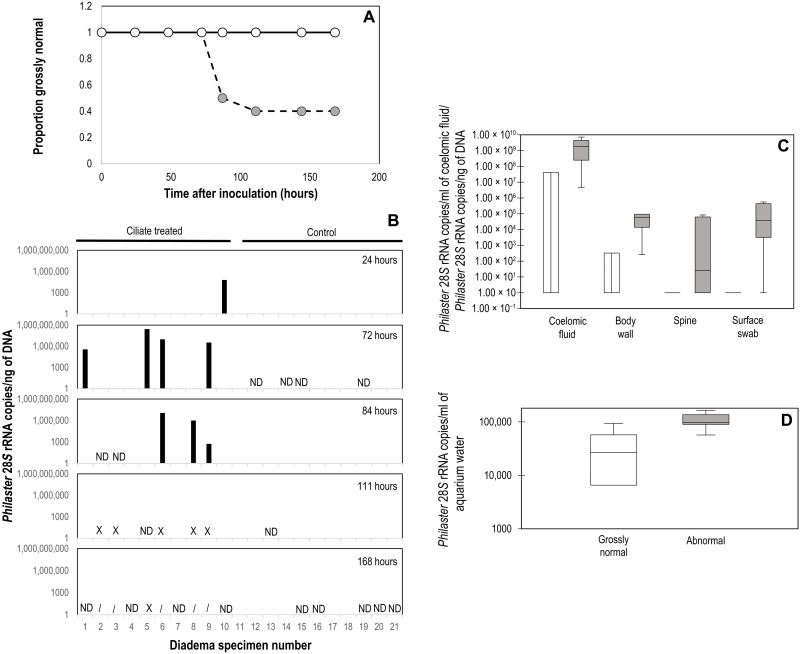
*D. antillarum* response to experimental ciliate challenge. (**A**) Proportion of observed grossly normal urchins in control (both <5-μm filtrate and DI water treatments; open circles) and ciliate-challenged treatments (gray circles) following experimental inoculation. (**B**) *Philaster*-like 28*S* rRNA abundance determined by qPCR in discarded spines of ciliate-treated or control urchins at 24, 72, 84, 111, and 168 hours after inoculation. ND, not detected; X, specimen terminated due to spine loss; /, specimens not available because of prior termination; blank indicates no lost spines. *Philaster*-like 28*S* rRNA abundance in dissected tissues of terminated specimens (**C**) and in >0.2-μm aquarium water particulate matter (**D**) of grossly normal (open box) and abnormal (gray box), ciliate-inoculated urchins. No ciliates were detected in body compartments of any of the control specimens by histopathology.

We then sought to experimentally challenge naïve juvenile *D. antillarum* (test diameters, 1.8 to 3.1 cm) with the cultivated ciliate in controlled aquarium studies to understand its pathogenicity. Twenty-one laboratory-reared *D. antillarum* (*24*) were placed individually into aerated 3.7-liter aquaria containing ozonated and mechanically filtered natural seawater and maintained at a constant temperature (26°C) throughout the experiment. Ten *D. antillarum* were amended with the ciliate culture (18 to 21 cells each) pipetted within 0.2 cm of their aboral surfaces, while five were amended with filtered (<5 μm) culture containing only bacteria and medium, and six were amended with deionized (DI) water. The animals were fed pellets (New Life Spectrum AlgaeMax Herbivore Diet) every 2 to 3 days, and each aquarium was subjected to a 50% water change every 2 days, with daily fecal pellet removal. Specimens were monitored daily for spine loss, loss of tube foot attachment, and spine movement/reaction to stimuli. Between 72 and 84 hours after inoculation, five ciliate-treated specimens lost >5 spines each (three specimens lost >20 spines) (Fig. 1D), and between 84 and 96 hours, an additional specimen lost >10 spines. Controls (both <5-μm filtrate and DI water) experienced no gross signs throughout the course of the experiment, but five lost single spines, presumably due to abrasion (Fig. 4). The *Philaster-*like ciliate was detected by qPCR in aquarium water of all ciliate-treated urchins at the termination of the experiment (168 hours) and was heavily enriched in the spine, body wall, and coelomic fluid of specimens that experienced spine loss. Ciliates with morphology consistent with Scuticociliata were observed in wet mount tissue of ciliate-treated urchins from the experiment during histological examination (see the Supplementary Materials). The ciliate was also detected by qPCR in lost spines of ciliate-challenged specimens that remained grossly normal, indicating that the ciliate can persist outside of the host in dead urchin tissues. The *Philaster-*like ciliate was not detected in any lost spines of control urchins. The pattern of *Philaster*-like ciliate occurrence suggests that early association occurs in tube feet, spines (which may be discarded after colonization), and spine bases, then in body wall tissues away from spine bases, and lastly in the coelomic fluid of abnormal specimens. None of the ciliate-challenged urchins from the experimental challenge study that remained grossly normal (*n* = 4) had observable ciliates by either tissue wet mount or histological sections, but two of six urchins that became abnormal during the experiment had ciliates in tissue wet mounts, and three had scuticociliates observed in histological thin sections.

## DISCUSSION

Considering observations from natural infections, the transmission potential demonstrated in the challenge study, and observations in culture, we suggest that the *Philaster*-like ciliate represents a highly prolific pathogen capable of invading tissues of grossly normal urchins and causing mass morbidity and mortality under conditions that are not yet well understood. Scuticociliates are implicated as causative agents in a variety of other diseases of marine vertebrates and invertebrates but are predominantly described in captive species (*25*, *26*). Adverse conditions affecting organismal immunity have previously led to scuticociliatosis in vertebrates, notably leopard and angel sharks in San Francisco Bay (*27*). Scuticociliates in the family Philasteridae typically cause skin lesions but may also cause massive systemic infection (*28*). Recently, *Philaster* spp. have been consistently found in association with several coral diseases, ingesting host cells (*18*–*22*, *29*) and potentially acquiring zooxanthellae from ingested corals (*20*). Ciliates have been observed in urchin digestive tracts as far back as 1932 (*30*, *31*), but *Philaster* spp. have not been reported in urchins of the Caribbean region. Furthermore, to the best of our knowledge, ciliates have never been observed in association with urchin diseases elsewhere (*14*, *32*–*34*). It is unknown whether this ciliate taxon is new to the region or if it is endemic but influenced by prevailing conditions to cause mass mortality. Scuticociliates are well known to occur under high-productivity conditions where they thrive on a wide variety of organic substrates (*35*). One hypothesis is that the *Philaster-*like ciliate experienced explosive growth under high-productivity conditions observed at several sites in the Caribbean in spring 2022, dispersed rapidly to other locations throughout the Caribbean, and then began to slow in the summer when conditions changed. However, more research is needed to understand the environmental conditions, host factors, and oceanographic processes driving infection dynamics at local and Caribbean-wide scales.

Microscopic and molecular detection patterns, as well as the fulfillment of Koch’s postulates by experimental challenge, strongly support our interpretation that the *Philaster*-like ciliate is the causative agent of *Diadema* mass mortality in the areas surveyed in this study. We term this mass mortality “*D. antillarum* scuticociliatosis (DaSc).” It is currently unknown whether DaSc was involved in the 1980s *D. antillarum* mass mortality. The association between DaSc and calm water ports and harbors, and the proximity of some sites where the condition initially occurred to river mouths, is of particular interest since these habitats may foster enhanced productivity of microorganisms in general (*15*). However, subsequent emergence at sites away from ports and harbors suggests that other host population characteristics or dispersal factors, potentially including currents, eddies, *Sargassum* rafts, migratory fish, or seabirds, may influence spread of the ciliate. The overlapping geographic ranges of both *D. antillarum* mass mortality and the stony coral tissue loss disease affecting numerous stony coral species in the southeastern region of the Dominican Republic in 2021 (*36*) are notable, since *Philaster* spp. are associated with coral diseases (*20*). However, it remains to be determined whether similar factors eliciting mass mortality occur between these hosts. It is also unclear whether the *Philaster*-like ciliate can affect other species of urchins or other species in the coral reef habitat in which *D. antillarum* occurs.

## MATERIALS AND METHODS

### Sample collection and processing

*D. antillarum* specimens were collected at 23 sites in nine jurisdictions in the northern and eastern Caribbean Sea to obtain transcriptomic, microbial, and histopathological data (table S1). In most jurisdictions, a reference site was chosen that was unaffected by the sea urchin mortality in addition an affected site with abnormal and grossly normal urchins. In the Turks and Caicos Islands, the condition was not observed during routine diver surveys (i.e., it was a reference site). Specimens from Brewer’s Bay, St. Thomas, USVI were collected in February 2022, frozen immediately in plastic bags, and stored until analysis. At all other sites, specimens (*n* = 3 grossly normal at reference site, *n* = 3 grossly normal at affected site, and *n* = 3 abnormal at affected site) were collected by scuba divers or snorkelers and transported to laboratory facilities for dissection. Abnormal specimens were collected when they exhibited early signs of the condition—e.g., drooping spines and falling over—but before spine loss occurred, with the goal of capturing acute processes contributing to mortality, as opposed to opportunistic infections triggered by tissue death. At some sites, surface sediments were sampled near collected urchin specimens by scooping into a sterile 50-ml centrifuge tube. At Saba, seawater was collected from near the aboral surface of all specimens using sterile 15- or 50-ml centrifuge tubes. In St. John, 250 ml of seawater was syringe-filtered onto a 0.2-μm filter on-site.

In the laboratory, coelomic fluid (0.5 ml) was drawn from all specimens using sterile syringes fitted with 22-gauge needles introduced through the peristomal membrane. Coelomic fluid was expunged into 2.5 ml of RNALater. Additional coelomic fluid (0.9 ml) was drawn into 1-ml syringes (22-gauge needle) prefilled with 0.1 ml of formalin for cytology (*37*) from specimens at Saba, St. John, and St. Croix. Spines (and spine bases; three per specimen) were removed using needle-nose pliers and placed into 3 ml of DNA/RNA Shield (Zymo Research). The urchins were then cut in half along the oral-aboral plane. Small pieces (approximately 5 mm by 5 mm) of body wall, gonad, and digestive tract were removed from one half with forceps and placed into vials containing RNALater. The remaining half of the urchin was preserved in Z-Fix (see the “Histopathological assessment” section below) for histopathological assessment (*38*). All specimens were transported to the laboratory at Cornell University for further analysis.

### Comparative transcriptomics of *D. antillarum* mass mortality

In two of the jurisdictions (St. John, USVI, and Saba, Caribbean, Netherlands), transcriptomes from coelomic fluid of grossly normal (*n* = 2) and abnormal (*n* = 2) individuals from an affected site and of grossly normal individuals from a reference site (*n* = 2) were prepared alongside grossly normal (*n* = 2) aboral body wall tissues from a reference site to examine expressed genes and microorganisms associated with infection (table S2). RNA was extracted from coelomic fluid (300 μl) and body wall tissues (200 mg) using the Zymo Quick-RNA Tissue/Insect Kit using on-column deoxyribonuclease I digestion per the manufacturer’s protocols. Transcriptome libraries were prepared from purified RNA extracts using the Zymo RiboFree kit and sequenced on a single run of Illumina MiSeq using a Nextera DNA Library Prep Kit at the Biotechnology Resource Center at Cornell University. All sequences have been deposited at National Center for Biotechnology Information (NCBI) Sequence Read Archive under accession numbers SRR22260770 to SRR 22260777 (table S3).

Sequence reads were initially trimmed of adapters and assessed for indices and quality (*N* ≤ 1; quality = 0.05) using CLC Genomics Workbench 8.5.1 (QIAGEN). Subsequently, all sequence libraries were assembled globally [0.8 of sequence identity, 0.5 of overlap, mismatch cost of 2, insertion cost of 3, deletion cost of 3, and minimum contig size, 500 base pairs (bp)] using the native de novo assembler in the CLC Genomics Workbench to produce a contig spectrum. Differential gene expression analysis was performed with the “Empirical Analysis of DGE” function in the CLC Genomics Workbench with common dispersion filter cutoff of 5.0 to identify DEGs when comparing grossly abnormal coelomic fluid to grossly normal coelomic fluid specimens from affected and reference sites. DEGs with >10-fold enrichment and with *P* < 0.05 [extraction of differential gene expression (EDGE) test] were selected for further analysis. Contigs matching these criteria were annotated using several approaches. First, contigs were subjected to BLASTx (*39*) against the nonredundant (nr) database at NCBI to identify putative function. Second, contigs were subjected to a conserved domain database search (*40*) at NCBI to identify conserved domains. Third, contigs were compared by BLASTn against the Silva database (*41*) of small (SSU) and large (LSU) rRNA subunits of both eukaryotes and prokaryotes to identify ribosomes. Last, nonribosomal annotations were searched within the Kyoto Encyclopedia of Genes and Genomes database (*42*) to identify gene ontologies.

We analyzed 16*S* rRNA genes present in the transcriptomes by comparing the globally assembled contig spectra against the Silva SSU database (release 138.1) by BLASTn and examined patterns of read recruitment between libraries (fig. S1). Contigs matching 16*S* rRNAs were examined by DECIPHER (*43*) for chimeric sequences; however, none were detected.

### Quantitative PCR of *Philaster*-like ciliate 28*S* rRNA

The contig matching the 28*S* rRNA of *P. apodigitiformis* (tDa_c52) was aligned against available 28*S* rRNAs from other ciliates identified by BLASTn against the nr database at NCBI using the MUSCLE algorithm (*44*). PCR primers (table S4) and an internal hybridization probe (i.e., TaqMan chemistry) were designed around a 120-nt region that was dissimilar to other *Philaster* spp. sequences using Primer3 (*45*). Primers were inspected for similarity to nontarget sequences by comparison against the nr database by PSI-BLAST.

DNA was extracted from coelomic fluid (100 μl) and tissue samples (aboral and ventral body wall, spine base muscle, connective tissue, gonad, and digestive tract; 50 to 200 mg each) using the Zymo Quick-DNA Tissue/Insect Kit, including extraction blanks (1 per every 96 extractions). Water samples taken in the field (10 to 50 ml; frozen at the site of collection) were thawed and then centrifuged at 5000*g* for 10 min to pellet cells, the supernatant was decanted, and DNA was extracted from the pellet using the Zymo Quick-DNA Fungal/Bacterial Kit. Some water samples from the USVI were collected by filtration onto 25 mm diameter 0.2-μm–pore size syringe filters. DNA was extracted from these by first allowing the kit-supplied “lysis buffer” to infiltrate the filter in the plastic housing for 10 min before this was expunged into a bead beating tube, after which DNA was extracted from the liquid according to the Zymo Quick-DNA Fungal/Bacterial kit protocols. Sediment samples (0.2 g) from a homogenized surface scoop (10 ml collecting the top ~0.5 cm) were processed using the Zymo Quick-DNA Tissue/Insect Kit kit and further purified using Zymo III-HRC columns. All DNA extracts were quantified by Pico Green fluorescence using an ABI StepOne qPCR machine as a fluorometer.

qPCR was performed in duplicate 25-μl reactions for each extracted sample. Each reaction contained 1× SsoAdvanced Universal Probes Supermix (Bio-Rad), 0.02 μl of 100 μM primer and probe, and 1 μl of extracted DNA template. Each sample run was compared against an oligonucleotide standard spanning the entire amplified region (100 bp), which was diluted over eight orders of magnitude, from 2.41 × 10^7^ copies to 2.41 copies per reaction. qPCR reactions were subject to an initial activation step at 50°C for 2 min and 94°C for 10 min, followed by 50 cycles of denaturation at 94°C for 10 s and annealing/extension at 56°C for 60 s in an ABI StepOne qPCR machine. Data were inspected for standard linearity (*R*^2^ > 0.98). The qPCR products from two reactions that yielded amplicons were ligated into the pGEM-T vector (Promega) and JM109 cells and then Sanger-sequenced to confirm that the amplicon corresponded to the predicted product sequence.

### Enrichment culture of *Philaster*-like ciliate

Coelomic fluid (0.5 ml) from two abnormal *D. antillarum* specimens collected near Key Largo, Florida, USA (24.9508^o^N, 80.4904^o^W) was withdrawn in the field using a sterile syringe and 22-gauge needle that was inserted through the peristomal membrane and expunged directly into 10 ml of cultivation medium [0.2-μm sterile-filtered seawater amended with yeast extract (4 μl/ml of a 0.1 g/ml, w/v solution) and one sterile white rice grain per 10 ml of medium]. The enrichment cultures were transported to the laboratory at Cornell University. After 24 hours, the cultures were inspected by light microscopy, yielding cells similar to those observed in preserved coelomic fluid drawn from field-collected urchins. After 48 hours, the initial enrichment culture from one of the urchins (FWC2; where scuticociliates were confirmed in tube feet wet mounts) was subjected to dilution-to-extinction culture on fresh media, which resulted in xenic culture of uniform cells at 1:100 dilution. Cultures were incubated at 26°C and subcultured every 24 to 72 hours on fresh media.

An experiment was initiated to examine the impact of *Diadema* tissue homogenates on FWC2 growth. Nine 15-ml sterile conical-bottom tubes containing 5 ml of culture media were amended with 0.25 ml of actively growing FWC2. A tissue homogenate was prepared by dissecting 1 g of aboral body wall tissue from a frozen reference site *D. antillarum* specimen from Puerto Rico and homogenizing this with 10 ml of sterile-filtered seawater using a mortar and pestle. Two milliliters of tissue homogenate was twice passed through a 0.2-μm polyethersulfone syringe filter (“*Diadema* extract”). Three incubations were inoculated with 0.25 ml of the *Diadema* extract, three incubations were inoculated with 0.25 ml of unfiltered *Diadema* tissue homogenate, and three incubations received no amendment (controls). The tubes were incubated in a plant growth chamber at 26°C on a 12-hour light:dark cycle. Scuticociliate cell abundance was measured at the experiment start, 1, 2, 3, 6, and 12 days after inoculation by counting cells in a 1-ml counting chamber under 50× light microscopy (fig. S4).

### Histopathological assessment

Veterinary histopathological and coelocytological assessments were performed on the same specimens as used for qPCR detection. For cytology, coelomic fluid samples were withdrawn from specimens before dissection following the approach of Work *et al*. (*37*). Coelomic fluid (~1 ml) was withdrawn using sterile 1-ml syringes fitted with 22-gauge needles that had been prefilled with 0.1 ml of formaldehyde or glutaraldehyde and then immediately mixed by flicking. Subsamples of this mixture (0.25 ml) were smeared/dried onto glass slides and differentially stained using DiffQuick before observation under light microscopy at ×100 magnification. For histopathology, urchin specimens were bisected along the oral-aboral plane, and half of each specimen was preserved in Z-Fix (ANATECH LTD) solution diluted with seawater, where they remained undisturbed for >48 hours before transport to the laboratory at Fish and Wildlife Research Institute (FWRI), St. Petersburg, FL, USA. Before shipping, most of the fixative was drained, and each sample was wrapped in fixative-moistened paper towels to prevent desiccation. Upon arrival in the FWRI laboratory, fixative was immediately reamended to the specimens. After initial gross observation, fixed tissue samples were rinsed under tap water and then decalcified in 10% EDTA solution for 4 to 22 days (mean, 9 days ± 4 SD; *N* = 90) following the method described by Francis-Floyd (*38*). Either before or after the tissue decalcification, tube feet located adjacent to gills were carefully excised under a dissecting scope (degraded feet were selectively chosen for the affected specimens, but any normal feet were chosen for healthy specimens) and examined for the tissue wet mounts. From each urchin, the following 10 slides were prepared from tissues: ambulacrum plate including ampullae, interambulacral plate including gonad, gills and neighboring tube feet, esophagus, small intestine including ring canal, large intestine, periproct rectum attached to the madreporite, axial organ, Aristotle’s lantern, and mouth area including modified tube feet. Slides were prepared by longitudinal section in both cross and arbitrary orientation. Tissues were embedded within paraffin blocks and sectioned at 4 μm in thickness using a rotary microtome. For juvenile samples used in the experimental challenge study, the bisected half portion of the whole specimen was processed with longitudinal sections in situ, rather than extracting individual organs. Slides were stained with Mayer’s hematoxylin and eosin and thionin stains. Slides were examined with a compound microscope and scored for the presence or absence of ciliates. Fisher’s exact test with *P* values (Bonferroni correction applied) was used to compare prevalence of the ciliates in three categorized wild urchin groups (i.e., abnormal urchins from affected sites, grossly normal urchins from affected sites, and grossly normal urchins from reference sites) that were tested separately by histology and by tube foot tissue wet mounts with statistical packages in R (version 4.2.2.) (*46*). 
