# Supplementary material for: A scuticociliate causes mass mortality of Diadema antillarum in the Caribbean Sea
Source: Sci Adv. 2023 Apr 19;9(16):eadg3200. doi: 10.1126/sciadv.adg3200 (PMC10115408; doi:10.1126/sciadv.adg3200)
Supplement: Supplementary file 1 — Figs. S1 to S4 Tables S1 to S4 [file sciadv.adg3200_sm.pdf]

Science Advances
AAAS

Supplementary Materials for

A scuticociliate causes mass mortality of *Diadema antillarum* in the Caribbean Sea

Ian Hewson *et al.*

Corresponding author: Ian Hewson, hewson@cornell.edu

Sci. Adv. **9**, eadg3200 (2023)
DOI: 10.1126/sciadv.adg3200

This PDF file includes:

Figs. S1 to S4
Tables S1 to S4

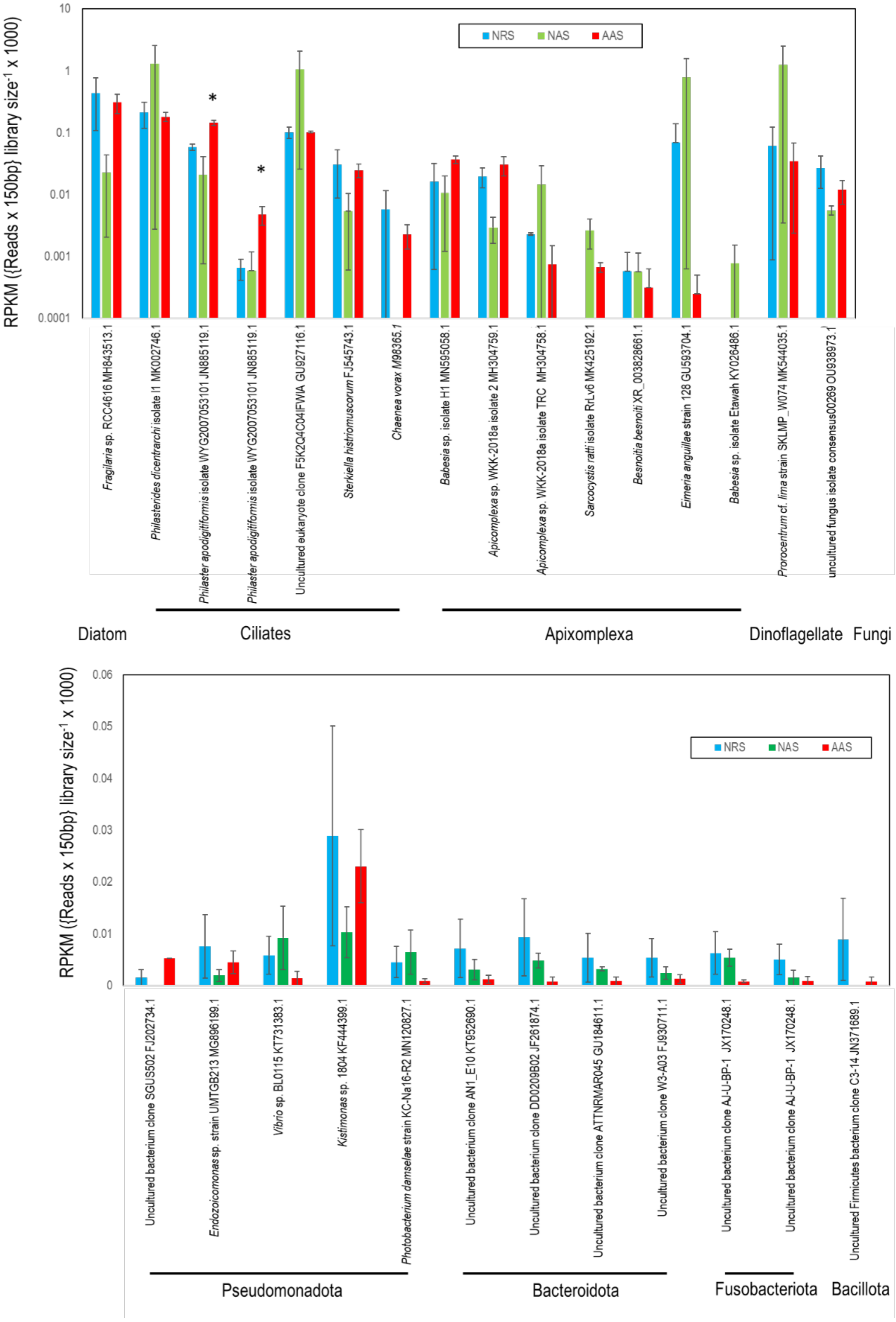

Fig. S1.

Recruitment of transcriptome library reads against contigs matching eukaryotic (top) and bacterial (bottom) ribosomal RNAs identified by BLASTn against the nr database at National

Center for Biotechnology Information (NCBI). NRS = grossly normal urchins from reference site, NAS = grossly normal urchins from affected sites, and AAS = abnormal urchins from affected sites. *Denotes significantly higher read recruitment from abnormal urchin libraries than in grossly normal urchin libraries from either affected or reference sites (Mann-Whitney U test).

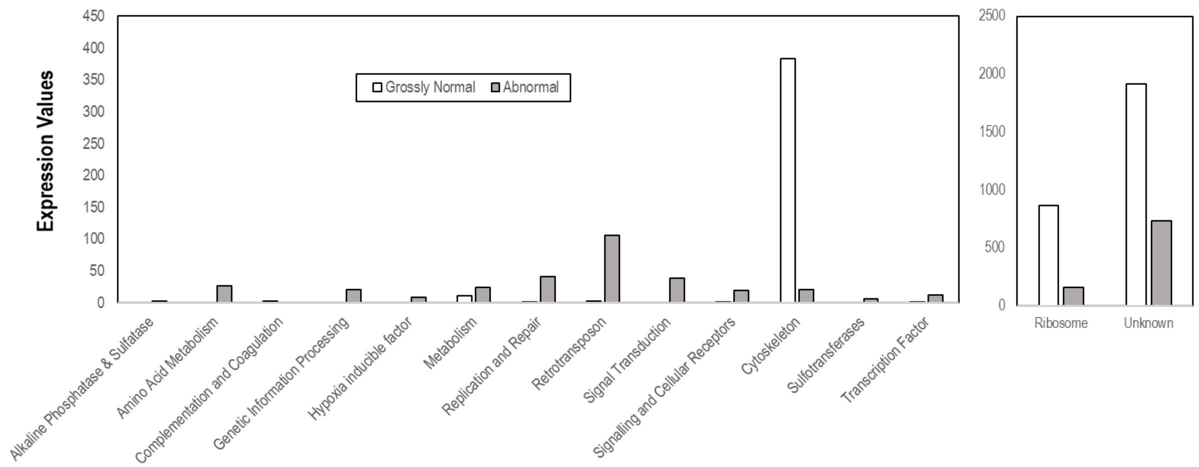

Fig. S2.

Aggregate gene expression values of annotated transcripts that were differentially enriched or suppressed in coelomic fluid transcriptomes prepared from grossly normal (at both reference and affected sites) and abnormal *Diadema antillarum*.

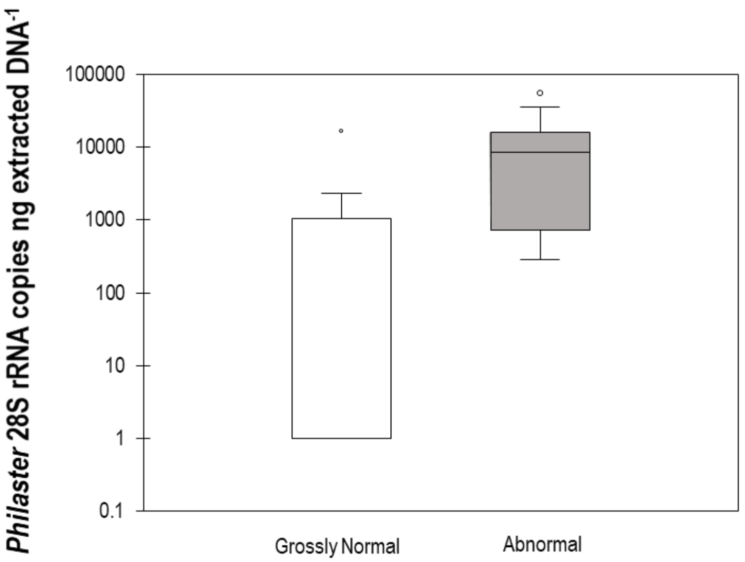

Fig. S3.

Quantitative PCR of body wall specimens of *Diadema antillarum* collected in Brewers Bay, St. Thomas Island (U. S. Virgin Islands), in February 2022. Because these specimens were frozen at -20ºC after collection, quantities of *Philaster* are not directly comparable to those collected in subsequent efforts which were preserved using RNALater. Abnormal urchins had significantly higher *Philaster* 28S rRNA copy loads than grossly normal urchins (Mann-Whitney U; $p = 0.017$; $n = 7$ abnormal specimens, $n = 8$ grossly normal specimens).

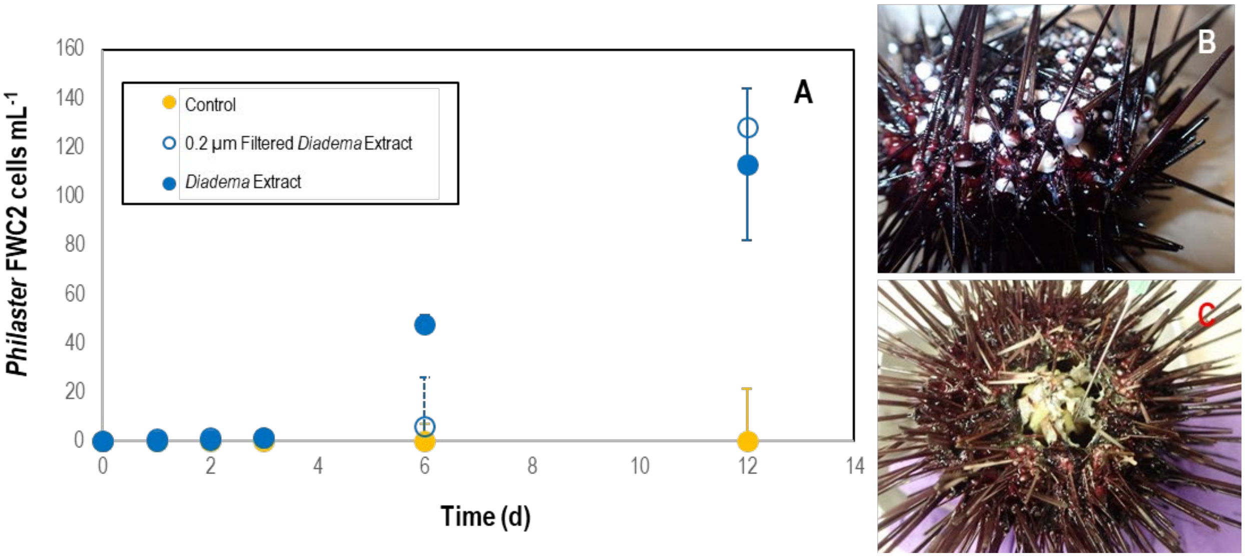

Fig. S4.

Growth of *Philaster*-like culture FWC2 in the presence of 0.2 µm-filtered and unfiltered *Diadema antillarum* tissue homogenate sampled at 0, 1, 2, 3, 6, and 12 d (A). The homogenate was prepared by grinding 1 g of *D. antillarum* body wall in 10 mL of sterile seawater. Photographs of abnormal urchin specimens FWC1 (B) and FWC2 (C) collected from near Key Largo, Florida, USA (24.9508°N; 80.4904°W), from which the *Philaster*-like ciliate was cultivated. Photo Credit: (B-C) William Sharp.

Table S1.

Collection location, date, and quantity of all *Diadema antillarum* specimens fixed for histological examination. Specimens were designated as grossly normal or abnormal in the field based on visual observations. Parentheses indicate the number of specimens for which *Philaster*-like ciliates were detected under a tube foot tissue wet mount examination (checked at post-fixation or post-decalcification of preserved materials); nc = tissue wet mount examination not conducted. Square brackets indicate the number positive for the *Philaster*-like ciliates under histological examination. *Specimens not exposed to ciliates during a transmission experiment. Site Cond. = Site Condition (A = Affected sites; R = Reference sites where no grossly abnormal *Diadema* were confirmed visually). U.S.V.I. = U.S. Virgin Islands; Carib. Netherl. = Caribbean Netherlands

**Origin**	**Date Collected**	**Site Cond.**	**Normal #**	**Abnormal #**	**Total #**
Three Moorings, St. John, U.S.V.I.	24 Mar 22	A	0	3 (3) [3]	3 (3) [3]
Brown Bay, St. John, U.S.V.I.	29 Mar 22	A	0	3 (2) [3]	3 (2) [3]
Three Moorings, St. John, U.S.V.I.	13 Apr 22	A	1 (0) [0]	0	1 (0) [0]
Pope Point., St. John, U.S.V.I.	21 Apr 22	R	3 (nc) [0]	0	3 (nc) [0]
Long Point., St. John, U.S.V.I.	22 Apr 22	A	3 (nc) [1]	4 (nc) [4]	7 (nc) [5]
Butler Bay, St. Croix, U.S.V.I.	30 Apr 22	R	3 (nc) [0]	0	3 (nc) [0]
Pull Point, St. Croix, U.S.V.I.	1 May 22	A	3 (nc) [3]	3 (nc) [3]	6 (nc) [6]
Saba, Carib. Netherl.	20 Apr 22	A	3 (0) [0]	3 (2) [3]	6 (2) [3]
	21 Apr 22	R	3 (0) [0]	0	3 (0) [0]
Boot Key, Florida, USA	4 May 22	R	3 (0) [0]	0	3 (0) [0]
Key West (Middle Ground), Florida, USA	6 May 22	R	3 (0) [0]	0	3 (0) [0]
Key Largo, Florida, USA	7 May 22	R	3 (0) [0]	0	3 (0) [0]
Miami, Florida, USA	12 May 22	R	3 (0) [1]	0	3 (0) [1]
Port Everglades, Florida, USA	14 May 22	A	2 (0) [1]	1 (1) [1]	3 (1) [2]
Key Largo, Florida, USA	15 June 22	A	0	2 (1) [2]	2 (1) [2]
Key Largo, Florida, USA	16 June 22	A	1 (0) [0]	3 (2) [3]	4 (2) [3]
Key Largo, Florida, USA	23 June 22	A	1 (0) [0]	0	1 (0) [0]
Cerro Gordo, Puerto Rico	13 May 22	R	3 (0) [0]	0	3 (0) [0]
Escambrón, Puerto Rico	13 May 22	A	3 (1) [1]	3 (3) [3]	6 (4) [4]
True Bay East, Grenada	13 May 22	A	3 (nc) [0]	3 (nc) [3]	6 (nc) [3]
Turks & Caicos Islands, Site A	21 May 22	R	3 (0) [0]	0	3 (0) [0]
Turks & Caicos Islands, Site B	23 May 22	R	3 (0) [0]	0	3 (0) [0]
Experimental challenge study	2 Jul 22	-	0	5 (2) [2]	5 (2) [2]
	3 Jul 22	-	0	1 (0) [1]	1 (0) [1]
	5 Jul 22	-	4 (nc) [0]	0	4 (nc) [0]
	5 Jul 22	-	2* (nc) [0]	0	2 (nc) [0]
**TOTAL**			56 [7]	34 [31]	90 [38]

Table S2.

Sampling locations, characteristics, and number of specimens collected for molecular investigation of *Diadema antillarum* mass mortality. Cond. = Specimen condition (NRS = Grossly normal at reference site; NAS = Grossly normal at affected site; AAS = Abnormal at affected site). Spec. = specimens. U.S.V.I. = U.S. Virgin Islands; Carib. Netherl. = Caribbean Netherlands.

Jurisdiction	Site Name	Latitude/Longitude	Cond.	Sample Date	# Spec.
U.S.V.I., St. Thomas	Brewers Bay	18.3407N, 64.9769W	NAS	16 Feb 2022	5
	Brewers Bay	18.3409N, 64.9769W	AAS	16 Feb 2022	5
U.S.V.I., St. John	Cinnamon Cay	18.3563N, 64.7565W	AAS	22 Mar 2022	2
	Pope Point	18.3451N, 64.6938W	NRS	21 Apr 2022	3
	Long Point	18.3324N, 64.6786W	NAS	22 Apr 2022	3
	Long Point	18.3324N, 64.6786W	AAS	22 Apr 2022	3
U.S.V.I., St. Croix	Butler Bay	17.7498N, 64.8924W	NRS	30 Apr 2022	3
	Pull Point	17.7645N, 64.6557W	NAS	1 May 2022	3
	Pull Point	17.7645N, 64.6557W	AAS	1 May 2022	3
Saba (Carib. Netherl.)	Tide Pools	17.6433N, 63.2186W	NRS	7 Apr 2022	2
	Diadema City	17.6147N, 63.2489W	NAS	7 Apr 2022	3
	Diadema City	17.6147N, 63.2489W	AAS	7 Apr 2022	3
USA, Florida	Boot Key	24.6649N, 81.0961W	NRS	4 May 2022	3
	Key West	24.4809N, 81.8844W	NRS	6 May 2022	3
	Key Largo	24.9508N, 80.4903W	NRS	7 May 2022	3
	Miami	25.3215N, 80.2066W	NRS	12 May 2022	3
	Port Everglades	26.0872N, 80.1026W	AAS	14 May 2022	1
	Port Everglades	26.0872N, 80.1026W	NAS	14 May 2022	2
	Key Largo	24.9508N, 80.4904W	AAS	15 Jun 22	2
	Key Largo	24.9508N, 80.4904W	NAS	16 Jun 2022	1
	Key Largo	24.9508N, 80.4904W	AAS	16 Jun 2022	3
	Key Largo	24.9508N, 80.4904W	NAS	23 Jun 2022	1
Antigua and Barbuda	-	17.0269N, 61.8915W	NAS	4 May 2022	10
		17.0038N, 61.7375W	AAS	4 May 2022	5
St. Eustatius, (Carib. Netherl.)	The Wall	17.4820N, 62.9884W	NAS	6 May 2022	5
		17.4816N, 62.9881W	AAS	6 May 2022	5
Puerto Rico	Cerro Gordo	18.4839N, 66.3396W	NRS	13 May 2022	3
	Escambrón	18.4684N, 66.0888W	NAS	13 May 2022	3
	Escambrón	18.4684N, 66.0888W	AAS	13 May 2022	3
Grenada	True Blue East	11.9975N, 61.7681W	NAS	13 May 2022	3
			AAS	16 May 2022	3
Turks and Caicos Islands	Site A	21.8230N, 72.1503W	NRS	29 May 2022	3
	Site B	21.8003N, 72.3539W	NRS	29 May 2022	3
Dominican Republic	Bayahibe	18.3729N, 68.8442W	NAS	25 Jun 2022	3
		18.3729N, 68.8442W	AAS	25 Jun 2022	3

Table S3.

Library characteristics and National Center for Biotechnology Information (NCBI) Sequence Read Archive (SRA) accession numbers for transcriptome libraries prepared from *Diadema antillarum* tissues. NRS = Grossly normal at reference site; NAS = Grossly normal at affected site; AAS = Abnormal at affected site. U.S.V.I. = U.S. Virgin Islands; Carib. Netherl. = Caribbean Netherlands.

Sample ID	SRA Accession No	Trimmed Library Size (reads)	Collection Location	Specimen Condition
tDaCF9	SRR22260777	440,092	Long Point, St. John, U.S.V.I.	NAS
tDaCF10	SRR22260776	332,718	Long Point, St. John, U.S.V.I.	NAS
tDaCF13	SRR22260775	481,420	Pope Point, St. John, U.S.V.I.	NRS
tDaCF14	SRR22260774	435,446	Pope Point, St. John, U.S.V.I.	NRS
tDaCF17	SRR22260773	557,710	Long Point, St. John, U.S.V.I.	AAS
tDaCF18	SRR22260772	796,626	Long Point, St. John, U.S.V.I.	AAS
tDaBW4A	SRR22260771	286,112	Diadema City, Saba, Carib. Netherl.	NAS
tDaBW5A	SRR22260770	393,128	Diadema City, Saba, Carib. Netherl.	NAS

Table S4.

Primers, probe, and oligonucleotide standard used to determine the abundance of *Philaster*-like ciliate 28S rRNA gene copies. [FAM] and [TAMRA] denote reporter and quencher dyes on the oligonucleotide probe, respectively.

**Oligonucleotide Name**	**Oligonucleotide Type**	**Nucleotide Sequence (5'-3')**
Phil_28S_F	Primer	TAGGGCAAGTCCTTGGAATG
Phil_28S_R	Primer	TGCCACATTTTATCCACAGC
Phil_28S_Pr	Probe	[FAM]CAGCGTACGCTGTTGCTGC[TAMRA]
Phil_28S_Std	Oligo Standard	TAGGGCAAGTCCTTGGAATGCAAAACAGCGTACGCTGTTGCTGCCAAAAGGG AAAGGGGTTAATATTCCTCTAGTCGGCTGTGGATAAAATGTGGCA
